# Timely support for promoting mental wellbeing among families with young children –an interview study exploring the experiences of multi-professional practitioners in Finland

**DOI:** 10.1186/s12875-023-02156-9

**Published:** 2023-09-23

**Authors:** Emilia W. E. Viklund, Anna K. Forsman, Johanna Nordmyr

**Affiliations:** Faculty of Education and Welfare Studies, Health Sciences, Åbo Akademi University, Strandgatan 2, 65100 Vaasa, Finland

**Keywords:** Mental health, Health promotion, Qualitative study, Primary care, Professionals

## Abstract

**Background:**

Childhood is a critical period for promoting mental wellbeing and previous research suggests that various family-focused mental health promotion and early prevention initiatives are effective. The aim of the study was to explore Finnish health and social care practitioners’ views and experiences of mental health promotion practice targeting families with young children.

**Methods:**

Individual semi-structured interviews with 14 practitioners representing various municipal services, faith-based and third sector organizations were conducted in 2021 and analysed using thematic analysis.

**Results:**

Various challenges and opportunities for supporting mental health related to both structural features of the health and social care landscape and the varying needs of families were identified. The lack of resources as well as the social stigma associated with mental health problems and with public welfare services, hindered proactive work approaches and timely support. However, low-threshold initiatives and adapted information to families as well as further training about mental health for practitioners together with multi-professional collaboration and teamwork were suggested as potential enablers for mental health promotion.

**Conclusions:**

The study highlights the importance of reaching families in a timely manner in order to promote mental wellbeing and prevent mental health problems. The findings, bringing to the fore the practitioners’ own experiences and views, suggest how current practice could be developed in order to safeguard mental health and wellbeing for all families with young children. The practitioners’ views and experiences are key components when building future sustainable and proactive health and social care services.

**Supplementary Information:**

The online version contains supplementary material available at 10.1186/s12875-023-02156-9.

## Background

Childhood is a particularly critical period for promoting mental health [1]. While positive relationships and a caring environment support mental wellbeing, dysfunctional relationships and milieus can act as risk factors, challenging children’s mental health as they grow [1, 2]. From a public health perspective, proactive work approaches, i.e. both promotion- (enhancing or maintaining health resources) and prevention-focused initiatives (preventing ill health before it occurs or at an early stage), are equally important in order to enhance mental wellbeing and combat mental health problems [3, 4]. Previous studies suggest that various family-focused and group-based programmes and initiatives to promote parenting, families’ socioeconomic situation and children’s health and well-being are effective in promoting mental wellbeing and preventing mental health problems in families with young children [5–7]. However, these programs/initiatives can only show positive outcomes in terms of mental wellbeing if they reach the families. It is therefore important to gather context-specific knowledge about the implementation of mental health promotion and prevention work and related potential facilitators and barriers within current practice.

### Supporting family mental wellbeing and preventing mental health problems in Finland

Finland is a Nordic country with a strong public sector with welfare services that promote health, education and a social safety net for all (key principles: universalism and equality) [8]. Internationally, Finland is regarded as a family-friendly country with universal publicly funded services emphasising child welfare and family wellbeing, including primary care services for parents and children, generous family benefits and a subjective right to childcare [9]. Maternal, child health clinics and school health services all play a key role in the Finnish preventive and health promotion work targeting families [10]. Additionally, public day care services, pre-schools, family centres and social services offer a wide range of support [10]. Non-profit or voluntary organizations, as well as private sector organizations, are also active in health promotion and early prevention in Finland, offering various activities and support. Therefore, preventive and health promotion work is carried out by persons representing various professions and several sectors collaborate [11, 12]. In Finland, primary health and social services, including health promotion and prevention, have been the responsibility of municipalities [13], but with recent reforms of health and social services the responsibility for these services has been reallocated to regional “wellbeing services counties” (districts) from 2023 [14]. Child and family welfare services are also being developed through the reform and projects such as the Future Health and Social Services Centres Programme and the Child and Family Service System Programme [15].

Even though proactive work approaches (mental health promotion and primary and secondary prevention) are currently emphasized in Finnish national legislation [13, 16] and policies [2] at different levels [17], previous findings [17, 18] highlight that mental health promotion and early prevention services for children are not currently working effectively. The aim of the current study was therefore to explore Finnish social and health care practitioners’ views and experiences of current mental health promotion practice for families with young children (under the age of seven).

## Methods

### Study design

A qualitative study design was used and semi-structured interviews underlies the study results. The framework for reporting qualitative studies (COREQ) [19, 20] was followed during the drafting of the article and a completed checklist is included as Supplementary material.

### Setting, participants and recruitment

The study is based on 14 individual interviews conducted in April-June 2021 in the Finnish Ostrobothnia region. Study information was circulated among regional health and social care organizations offering services and activities targeting parents of, or families with, under school-aged children. Practitioners currently working with families with young children were eligible for study participation. Participants were self-selected and all of the study participants gave their informed consent to participating in the study as well as to recording prior to initiating the interviews.

All informants were professionals working with parents of, or families, with young children. Some informants primarily focused on supporting families facing various challenges, i.e. from a theoretical perspective representing a preventive, risk-focused practice while others represented universal, health promotion work where sub-groups of parents/families are not singled out. The participants represented various municipal health and social care services (*n* = 7), two faith-based organizations (*n* = 3), and three third sector organizations (*n* = 4). All study participants were women and their experience in working with families with young children varied between four and twenty years (mean 9.4 years).

### Data collection

The interviews were semi-structured, utilizing an interview guide (see Supplementary material) encompassing several broad questions focusing on the informants’ work in supporting mental health and wellbeing among families with young children and related multi-professional and intersectoral collaboration. The interview guide was developed by the researchers specifically for the current study and has not previously been published. The interviews, conducted online due to the covid-19 pandemic and related restrictions and lasting between 37 and 56 min, were recorded and subsequently transcribed (145 pages in total).

### Data analysis

Thematic analysis described by Braun and Clarke [21] guided the inductive and exploratory data analysis. The six step process includes data familiarization (1), raw data coding (2), searching for patterns among the codes and generating themes (3), reviewing the codes and themes (4), defining the themes (5) and the writing of results (6). Thematic analysis is not a linear process, but rather a constant movement between the analysis steps. The first author led the analysis process and the other authors critically reviewed the drafts of the results, which was revised several times. The analysis process is illustrated in Table 1.

**Table 1:** Illustration of the thematic data analysis process

Raw data	Notes	Codes	Sub-themes	Main themes
I4: […] Then it like also becomes an ethical conflict for me. Like, what do I do? Should I dedicate time to many… and not give enough to anyone, or should I dedicate enough time to a few and leave the rest without support? It was impossible and it tore at me terribly, this ethical conflict. […]. But now I have opted to support those that I see as much as I can	Not being able to support all is experienced as an ethical conflict among the practitioners	The supply–demand imbalance is influencing mental wellbeing at work	Resources in public services – challenging proactive and collaborative work approaches as well as professional wellbeing	Interacting within a complex system
I13: […] But families may not really be aware of what support they can receive from us. Child protective services are often associated with a very negative connotation, it’s like “oh no, they’re coming to take our children”. They don’t consider us a part of this [promotion and early prevention work], that we want to help. Our aim is to help and support the family so they can manage on their own. But the negative connotation is still there. So they don’t want anything to do with child protective services or social services for families	The prejudices regarding child protection services influences families picture of child protection services and can function as a barrier for seeking support	Stigma associated with child protection services challenging reaching families early on	Social norms and stigma among families with young children – inhibiting timely support activities from reaching families	Connecting with diverse families

## Results

### Reaching the families in need of support in time—challenges and enablers for supporting mental health among families with young children

**Fig. 1:**
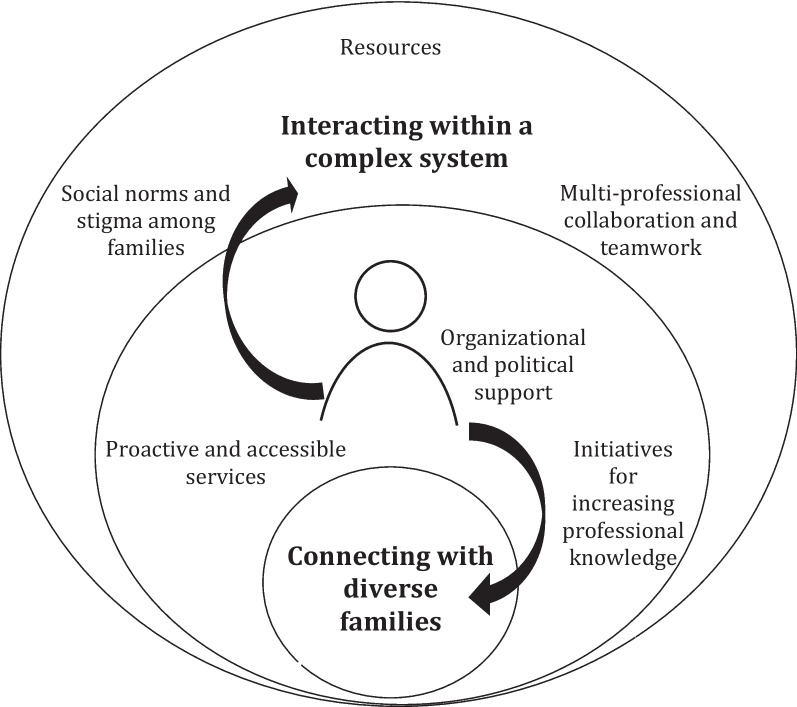
Illustration of the study findings

Supporting the mental health of families was seen as a vital part of the informants’ work descriptions, but the data analysis highlights that the current mental health promotion and prevention practice is influenced by various circumstances. Two main themes were generated from the thematic data analysis, each main theme including three subthemes (see Table 2). An illustration of the key study findings is presented in Fig. 1. The data was not analyzed separately for the informants representing different sectors; however, important nuances and differences were observed during the analysis process, which are noted in the presentation of the findings.

**Table 2:** The themes generated from the data analysis describing the study informants’ experiences of mental health promotion and early prevention practice among families with young children

Overarching theme	Main themes	Sub-themes
Reaching the families in need of support in time—challenges and enablers for supporting mental health among families with young children	Interacting within a complex system	Resources in public services – challenging proactive and collaborative work approaches as well as professional wellbeing Organizational and political support—enabling promotion and prevention work Multi-professional collaboration and teamwork—a challenge and an enabler within stagnant structures
	Connecting with diverse families	Social norms and stigma among families with young children – inhibiting timely support activities from reaching families Initiatives for increasing professional knowledge – a key for promoting mental wellbeing Proactive and accessible services – measures for lowering the threshold for families even further

### Interacting within a complex system

This first main theme captures circumstances related to the organization of current mental health promotion and early prevention practice, which could challenge or enable the informants’ work.

#### Resources in public services- challenging proactive and collaborative work approaches as well as professional wellbeing

The practitioners working within the public social and health services targeting families described the current work situation as a vicious cycle, where families with more comprehensive support needs were being prioritized and primary prevention and promotion work consequently left aside. The cycle was maintained by resourcing issues. Thus, the practitioners perceived that they were unable to practice mental health promotion and early prevention to the extent or in a way that they found necessary:

I5: [..].We have a personnel shortage, we have a queue, we have twenty or thirty families in a queue. We have been understaffed for a long time and we still are. This year has been extra turbulent in this regard so […]. The personnel shortage and resourcing leads to us not being able to do this the way we want to.

The practitioners suggested that an increased emphasis on primary prevention and mental health promotion in their daily work, would allow for some critical problems to be prevented and the queue of families in need of support to be shortened and resources released. However, this vicious cycle was perceived as challenging to break within current practice, marked by the limited resources:

I3: […] the personnel is understaffed in relation to the population size, which makes it, we could work in much more of a preventive manner and much more, like targeting the general public and perhaps preventing people from needing to come here for individual or family support. If they could come in an earlier stage. Also according to the social services act, that the family counselling services are under, the emphasis should be on prevention. And there is a wish for us to step in earlier in the families situations, but there are these resourcing challenges.

Additionally, the experienced supply–demand imbalance, resulting from a lack of resources, was not only seen as challenging the situation for families– it was also described as influencing the informants’ work performance and wellbeing:

I4: […] Then it like also becomes an ethical conflict for me. Like, what do I do? Should I dedicate time to many… and not give enough to anyone, or should I dedicate enough time to a few and leave the rest without support? It was impossible and it tore at me terribly, this ethical conflict. […]. But now I have opted to support those that I see as much as I can.

#### Organizational and political support—enabling promotion and prevention work

Having the trust of the management in one’s professional competence was considered an important prerequisite for practice and for offering families appropriate services. Hence being able to “call the right shots” — to decide upon what forms of support the families need based on their own professional competence, facilitated mental health promotion and early prevention practice as well as promoted wellbeing at work:

I5: […].That we have the freedom to make the best of the situation is an asset. I have a freedom as a professional to decide together with the clients what we do, how long the period is, what we are trying to achieve.

I9: Really, the support and trust of the manager is important. If it were not for that, the work would be quite daunting, no matter how great the work is and regardless of the feedback from the parents and families. […].

Additionally, the practitioners called for increased political support in terms of more economic resources for safeguarding mental health promotion and early prevention practice in the future — however economic decisions taken by the municipality, larger wellbeing services counties and the government were perceived as difficult to influence:

I3: Perhaps it is more about the political support, to see that it implies actual resources, investing in child and family mental health. […]. You are sort of missing that support, for that to happen, but it’s also an issue which is not easy to influence. Of course we raise the needs within the organizations and so on but..

#### Multi-professional collaboration and teamwork—a challenge and an enabler within stagnant structures

The communication and collaboration between different practitioners and various organizations were seen as crucial in order to reach families and offer timely support. However, the practitioners highlighted that multi-professional collaboration should be carried out systematically and within various levels of organizations and across sectors and that the collaboration should not be dependent upon and only connected to specific persons working within the organizations. From the practitioners’ point of view, forming multi-professional teams was seen as an ideal:

I12: Every person working with persons who experience mental health problems should work in a team. […]. There is a lot more strength in working in a team. Bringing things up in a team. And to some extent this is done already, but it’s not like”now X and I are meeting this family”,. You don’t have that strength, our system is so vulnerable in a way […]. But you would have to build a system where there is maybe teams with different professions, over the whole field. I would like to see that.

Even though multi-professional collaboration was identified as best practice, the implementation of such work approaches was not without difficulties, related to structures within the organizations and the municipalities that challenged the ability to systematically work in multi-professional teams and across sectors:

I1: I think, now I’m complaining, I think the implementation of the model is falling short, because they school us in new programs and we’ve attended courses and taken this to heart and thought” ok, we are working in line with this because it’s what you decided”. But then they haven’t, how should I put it […]. We can’t work in line with the new model, not in my municipality, and I see it falling also in other municipalities, so I think that’s really bad.

Information sharing between organizational units or between professionals representing different organizations was described as a core factor for family outreach, but insufficient communication between units and organizations was perceived to be a bottleneck, potentially hindering multi-professional collaboration and by extension proactive work approaches:

I13: The mother may gets tips and advice from a contact and the dad gets from theirs and then they receive third party information from the team responsible for the younger children. That’s maybe where it fails. The ideal would be that there would be some kind of, I don’t know, where the whole family receives the support they need. So there is an exchange of information, and a holistic picture of the family.

### Connecting with diverse families

The second main theme captures factors related to the interaction between the practitioners and the families they meet and work with.

#### Social norms and stigma among families with young children—inhibiting timley support activities from reaching families 

Practitioners working within mostly the third sector organizations described challenges related to service reach. The practitioners experienced that particularly social activities do not reach the families who would perhaps benefit from the activities the most. Thus, universal health promoting activities aiming to increase e.g. peer support may not reach all groups of families:

I2: […]. And in my experience perhaps it’s, well, those with higher education maybe? Those who already have a lot of information and for whom it’s easy to read information and maybe have supportive networks already, they find these forums more easily.

Outdated misconceptions among parents regarding the service options provided by the social and child protection service were seen as a barrier for parents taking advantage of support intended to promote a smooth functioning of family life regardless of eventual problems. They were also considered to influence the tendencies for seeking support and guidance early on. Common misconceptions portray the service receivers as bad parents and many parents seem to believe that the enrollment in child protection services automatically postulates a risk of children being taken into care outside their homes – a scenario and label that many parents feared:

I13: But families may not really be aware of what support they can receive from us. Child protective services are often associated with a very negative connotation, it’s like “oh no, they’re coming to take our children”. They don’t consider us a part of this [promotion and early prevention work], that we want to help. Our aim is to help and support the family so they can manage on their own. But the negative connotation is still there. So they don’t want anything to do with child protective services or social services for families.

#### Initiatives for increasing professional knowledge – a key for promoting mental wellbeing

The practitioners perceived that their work is increasingly focused on promoting mental wellbeing and preventing mental health problems, but they also encounter mental health problems among clients. Therefore, opportunities for continued education was perceived as important and a key factor for work motivation and wellbeing:

I5: In order to develop, all employees need continual education, in relation to current events and working methods in society. The world is constantly evolving and even though you have an education you need this, in order to be inspired and have confidence in yourself, but also for the knowledge it’s crucial. And that’s something I can say, now that I’m not far from retiring and look back on the courses I’ve attended, they have been absolutely crucial […].

Overall, knowledge regarding mental health and early signs of mental health problems was perceived to be essential for being able to identify the need for support in a timely manner. Without knowledge, early signs of mental health problems might be missed and therefore, every practitioner within social and health services need to have knowledge regarding mental health:

I12: A lot of people come to the health centres with diffuse problems, they may not receive a diagnosis, however it may be a sign of something else. It is perhaps easier with measurable things than those that are more “I have a stomach ache because I am worried about something”. Maybe they [other professionals in primary health care] should receive more education.

#### Proactive and accessible services – measures for lowering the threshold for families even further

The importance of the availability of low-threshold services for families was addressed during the interviews. One example was an early prevention initiative aiming to lower the threshold for support-seeking early on by addressing the social stigma associated with social and child protection services. The initiative constituted an opportunity to receive professional support with everyday life challenges from a family worker without having to be enrolled as a client in the social and child protection services nor having to sign up for a longer period. The practitioners were optimistic about such initiatives and requested more of such low-threshold services for families:

I4: And in this municipality they have recently initiated this service […]. With a low threshold you can contact a family worker that visits the family and can assist where needed. This is quite new. I think it’s a great initiative, and I suspect a lot more of it would be needed.

Also reflecting practical measures related to accessibility, moving some of the group-based activities and low threshold informational events online were also suggested to reach more families. Existing regional Facebook groups for families with young children were also seen as usable forums for spreading information along with having accessible websites. The potential with online meetings for reaching families, especially in rural areas, was recognized:

I8: This is the positive side of the online dimension, not having to be in a certain municipality to participate. Earlier it was in a [specific location in specific municipality] and then it’s much more difficult to participate if you don’t actually live in that community.

## Discussion

This study explored Finnish practitioners’ experiences of mental health promotion and early prevention work targeting families with young children (under the age of seven). The findings highlight the perceived importance of reaching families in a timely manner, before the development of mental health problems and related social challenges accumulate. The practitioners participating in the study experienced various challenges in their work to support mental health of families. These were related to the complex health and social care systems and organizations, and to connect with families with diverse needs. They also suggested enabling factors and opportunities for developing future practice to better meet the diverse needs of families and the complex and changing social and health service and societal landscapes. Thus, the study findings may provide insight into why mental health promotion for children and their families previously has been perceived to not work optimally from a professional perspective [6, 17, 18].

The work of practitioners within the public sector was perceived to be affected by limited resources, which in turn influenced the ability to work proactively to prevent mental health problems and promote mental wellbeing. Previous studies have highlighted that personnel targets for child mental health services in Finland are not currently being met, which affects both the quality and the service supply, and more resources for services aimed at promoting family wellbeing are being requested [17]. The practitioners in this study described an imbalance between the needs of families and the availability of services, which meant that families with more complex support needs were often prioritized and primary prevention and promotion work given low priority. Hence, a vicious cycle that was considered difficult to break.

While the practitioners working in public social and health services struggled with implementing proactive work approaches in practice, the informants who worked with universal health promotion activities, e.g. organizing social activities and support groups, highlighted difficulties related to outreach and especially regarding reaching families representing diverse backgrounds and family situations. The practitioners were concerned that families in a more advantaged socio-economic positions seem to participate in the universal health promotion activities they offered to a higher extent than less advantaged families, which could further widen health gaps and increase polarization. Despite large investments in inequality reducing efforts, health inequalities persist in the Nordic countries [22]—and it is argued that the health gaps are even greater than those observed in southern European countries [23].

The European Commission [24] recently launched a comprehensive approach to strengthening mental health in a changing Europe. This approach highlights the importance of equal and timely support to promote mental wellbeing and prevent and care for mental health problems [25]. Thus, in addition to emphasizing the need to be proactive in addressing mental health issues, the comprehensive approach also addresses the importance of working with structural factors and the social determinants of mental health. Studies have addressed that health promotion initiatives, such as parenting programs, do not sufficiently address the social gradient in health [26]. Therefore, more studies need to explore how health inequalities are currently being addressed in mental health promotion work [27] and how they can be more effectively considered in the future.

One work approach that potentially could reduce health inequalities by applying a holistic approach to health and its determinants is social prescribing. Social prescribing refers to a work process that links clients mostly in primary care with services and activities in the community through referrals from health professionals [28]. By strengthening collaboration and communication between practitioners and across sectors, this type of organized multi-professional collaboration has the potential to overcome some of the barriers identified in this study, such as poor communication and lack of organizational structures. The pathways and models for multi-professional collaboration offered by social prescribing can thus potentially enable good practice and ensure that different types of support reach a diverse group of families in order to promote mental wellbeing. Intersectoral collaboration and partnerships are at the heart of modern health promotion practice [4] and a perquisite for providing families with appropriate, timely and person-centered support [29, 30]. However, the current evidence base on social prescribing is inconclusive and scattered [31] and more studies are needed to explore whether and how social prescribing could support the mental wellbeing of families in different contexts.

Overall, initiatives to lower the threshold for services aimed at promoting mental wellbeing and preventing mental health problems are welcomed by practitioners in this study. The importance of proactive communication and easily accessible information to reach families with diverse needs was highlighted in the study findings. For instance, online-based services, activities and information were suggested to support the availability of services. Knowledge, low-threshold services and accessible information are also important strategies for combating the negative stereotypes and social stigma associated with mental health-related challenges, which have been identified in previous studies as barriers to help-seeking [32, 33]. A national survey [34] shows that parents who had trouble coping with daily life were the least likely to report their need for support to professionals. Social stigma may be a reason for reluctance to seek professional help for mental health-related challenges. Parents in a study by Sayal et al. [35] described being concerned that they would be judged as poor parents if they sought professional support. The informants in this study also experienced that preventive services provided by public social and child protection were associated with outdated misconceptions about their forms of support, which in turn was perceived to challenge their ability to implement proactive work approaches. The stigma associated with these types of public welfare services not only has a negative impact on service outreach but has also been associated with negative mental health outcomes and high staff turnover among practitioners [36, 37].

The strong tradition of dysfunction-orientation in health organizations has been suggested as an additional challenge to the implementation of health promotion and prevention work by practitioners in previous studies [38, 39] as non-medical interventions and proactive approaches have not been valued as much as treatment and care. As we are facing uncertain economic times, it would be important to refrain from cutbacks in mental health promotion and early prevention efforts targeting families. Service cuts in this area during the economic recession in the early 1990s in Finland led to an increased need for mental health services among children, which in the end are more resource intensive for the society than the costs of mental health promotion and early prevention efforts [40].

The experiences and views of practitioners presented in this study can support the development of mental health promotion and early prevention endeavors. In addition to the practitioners’ perspective presented in this study, it would be important to hear the families’ own voice in future studies. As the Finnish social and health service landscape is currently changing due to ongoing social and health service reforms [14] this may be a particularly good time to actively involve both practitioners and service users in shaping future social and health services. Empowerment and participation are central principles of mental health promotion [41] and the expertise and contextual knowledge of practitioners and families should be seen as key to building sustainable social and health systems, including services with a focus on mental health promotion and early prevention.

### Strengths and limitations

There are some limitations that need to be considered in relation to this study. Firstly, the researchers are central to the whole research process, thus, research is not free from influences of time and context, nor the theoretical underpinnings of a study and the experiences of the persons conducting the analysis [21]. The fact that the authors have research interests and experience with other studies focusing on mental wellbeing and health promotion is likely to have influenced the design of the interview guide and the analysis of the data. The pre-defined questions in the interview guide are an additional limitation with the present study [42] as they are subject to the pre-understanding of the authors. On the other hand, the questions were broad in nature allowing informants to freely share their views, while also ensuring the coherence of the data material. Furthermore, it is always important to be aware of the power imbalance between participants and researchers, which may lead to the participants discussing topics that they would not otherwise have mentioned. It is also important to note that the interview guide was not pilot tested prior to conducting the interviews. However, after the first interview was conducted the authors discussed the interview guide and there was an opportunity to change or develop the questions if they were not perceived to be appropriate.

It is furthermore important to highlight that the interviews were conducted online due to the Covid-19 pandemic. On the one hand, online interviews enabled the participation of practitioners and ensured their safety and health by avoiding face-to-face contact, which was in line with the health authority guidelines at the time. On the other hand, the online format may have influenced the reliability of the study, including the interaction during the interviews. Previous studies have reporter that the online format can reduce the richness of interview data [43]. Additionally, the pandemic is likely to have influenced the findings themselves. For instance, the pandemic may have affected the provision of services in third sector organizations and put additional pressure on public sector practitioners.

Overall, the study informants, data, procedure and the data analysis are described in text, tables and figures in order to increase study transparency and trustworthiness. However, a further collaboration with the study informants in e.g. receiving feedback about the findings could have enhanced trustworthiness even further. One of the main study strengths is the multi-professional approach, as it contributed to rich data material on the practitioners’ views and experiences of mental health promotion and early prevention among families with young children.

## Conclusions

Timely and equitable support to promote mental wellbeing is highly important for families with young children. However, social and healthcare practitioners perceived that, due to rigid structures within social and health organizations and the social stigma associated with mental health problems and related services, mental health promotion and prevention services do not currently reach families in a timely manner and/or reach as many families as they could. In order to improve current practice and health and social care services alike, there is a need to lower the threshold for parents to seek professional psychosocial support and to explore new ways to facilitate the collaboration between different practitioners across sectors who encounter families in their work. At the societal level, there is also a need to increase knowledge about mental health and related initiatives, and to allocate more resources to mental health promotion and early prevention. Overall, this kind of context-specific knowledge based on practitioners’ own experiences and views is key to building sustainable social and health systems and should be increasingly taken into account.

### Supplementary Information

**Additional file 1: Supplemental material. **COREQ 32-item checklist for interviews and focus groups (Tong, Sainsbury & Craig, 2007).

**Additional file 2: Supplementary material. **Interview guide (translated to English from Swedish/Finnish by the authors).

## Data Availability

The dataset underlying this article is not publicly available due to stipulations in the informed consent, thus the study participants not giving consent for their data to be shared publicly. However, the data are available from the corresponding author on reasonable request.
