# Supplementary material for: Timely support for promoting mental wellbeing among families with young children –an interview study exploring the experiences of multi-professional practitioners in Finland
Source: BMC Prim Care. 2023 Sep 23;24:196. doi: 10.1186/s12875-023-02156-9 (PMC10517518; doi:10.1186/s12875-023-02156-9)
Supplement: Supplementary file 1 — Additional file 1: Supplemental material. COREQ 32-item checklist for interviews and focus groups (Tong, Sainsbury & Craig, 2007). [file 12875_2023_2156_MOESM1_ESM.docx]

**Supplemental material**

COREQ 32-item checklist for interviews and focus groups (Tong, Sainsbury & Craig, 2007).

| **Research team and reflexivity** |  |  |
| --- | --- | --- |
|  | Interviewer/facilitator | Author 3 conducted most of the interviews together with a project assistant and Author 2 conducted one of the interviews. |
|  | Credentials | Author 1= PhD  Author 2 = DrPH  Author 3= PhD |
|  | Occupation | Author 1 = Postdoctoral researcher  Author 2= Associate Professor  Author 3= University lecturer |
|  | Gender | All of the researchers are female. |
|  | Experience and training | Author 1 = Has experience from planning and conducting several interviews.  Author 2= Has experience from planning and conducting multiple interviews and led national and international research projects on health promotion contexts and approaches.  Author 3= Has experience from planning and conducting multiple interviews. |
| **Relationship with participants** |  |  |
|  | Relationship established | The researchers were not employed by any of the organizations that the practitioners represented. The recruitment of informants report no bias grounded on dependency issues. |
|  | Participant knowledge of the interviewer | All of the informants knew which organizations the researchers represented and their research interests in mental health promotion. |
|  | Interviewer characteristics | In the manuscript strengths and limitations section, the researchers’ preunderstandings as health promotion scientists are highlighted as a potential bias. |
| **Theoretical framework** |  |  |
|  | Methodological orientation and theory | Thematic analysis with an explorative and inductive approach. |
| **Participant selection** |  |  |
|  | Sampling | Purposeful sampling, self-selection. |
|  | Method of approach | Study information was circulated among regional organizations offering services and activities targeting parents of, or families with, under school-aged children. |
|  | Sample size | 14. |
|  | Non-participation | Information about the study was sent by email to organizations focused on mental health promotion and prevention work among families. The study information was spread within the organization to practitioners. About 23 organizations were contacted. |
|  | Setting of data collection | The interviews were all conducted online due to the COVID-19 pandemic. |
|  | Presence of non-participants | - |
|  | Description of sample | The participants represented various municipal services, faith-based organizations, and third sector organizations. All study participants were women and their work experience varied between four and twenty years. |
| **Data collection** |  |  |
|  | Interview guide | A semi-structured interview guide encompassing broad themes and related questions guided the interviews. The interview guide was not pilot tested, but was discussed within the research group after the first interview. |
|  | Repeat interviews | - |
|  | Audio/visual recording | The interviews were audio recorded. |
|  | Field notes | The interviewers made notes during the interviews. |
|  | Duration | The interviews lasted between 37.55 and 56.28 minutes. |
|  | Data saturation | Data saturation was discussed among the researchers after the 13th interview and the researchers decided to not recruit more informants than already appointed. |
|  | Transcripts returned | - |
| **Data analysis** |  |  |
|  | Number of data coders | Author 1 conducted the initial coding of the data and all authors contributed to generating the themes. |
|  | Description of the coding tree | Table 2 displays the codes, sub-themes, main-themes and the overarching theme and Figure 1 is an illustration of the findings. |
|  | Derivation of themes | Two examples of the data analysis process are given in Table 1. |
|  | Software | Microsoft Word |
|  | Participant checking | - |
| **Reporting** |  |  |
|  | Quotations presented | The reporting of the findings include quotations from the raw data file (translated from Swedish and Finnish to English by the authors). |
|  | Data and findings consistent | The authors have made an effort to keep the findings close to the original (manifest) data and the authors who conducted the interviews recognized the discussions in the final results presentation. |
|  | Clarity of major themes | The major themes are described in text as well as in Table 2 and Figure 1. |
|  | Clarity of minor themes | The minor themes are described in the text as well as in Table 2 and Figure 1. |
