# Supplementary material for: Timely support for promoting mental wellbeing among families with young children –an interview study exploring the experiences of multi-professional practitioners in Finland
Source: BMC Prim Care. 2023 Sep 23;24:196. doi: 10.1186/s12875-023-02156-9 (PMC10517518; doi:10.1186/s12875-023-02156-9)
Supplement: Supplementary file 2 — Additional file 2: Supplementary material. Interview guide (translated to English from Swedish/Finnish by the authors). [file 12875_2023_2156_MOESM2_ESM.docx]

**Supplementary material**

**Interview guide (translated to English from Swedish/Finnish by the authors)**

**Introduction**

Could you tell me/us a little bit about yourself and your daily work?

**Mental health of families**

In your professional experience, what are the mental health and wellbeing needs of families with young children today?

**Mental health promotion work with families**

Do you feel that the work with supporting the mental health of families with young children can be carried out as it should be? If not, why?

Do you feel that you and your organisation are reaching the families you want to or should be reaching? If not, why?

Do you see any other development opportunities or needs related to your work that you would like to highlight?

**Work approaches related to mental health promotion**

Can you tell me/us about the measures and tools you use in your work to support the mental health of families with young children?

From your perspective, has the work of supporting the mental health of families with young children changed over time? If so, in what way?

**Collaboration in mental health promotion work**

Do you work together within your organization to support mental health among families with young children? If so, in what way?

What advantages and/or disadvantages do you see in working together to promote mental health?

Does your work to support families involve collaboration with other organisations? If so, how do you perceive this collaboration?

Are there any (other) organizations you would like to work with? If so, with whom and why?

**Support in the professional role**

What kind of support do you have and find valuable in relation to your own work?

Would you like any other / additional support? If so, what type of support would be valuable to you?

Is there anything else you expected us to talk about today that we haven’t covered?
